# Microbiota independent effects of oligosaccharides on Caco-2 cells -A semi-targeted metabolomics approach using DI-FT-ICR-MS coupled with pathway enrichment analysis

**DOI:** 10.3389/fmolb.2022.968643

**Published:** 2022-10-24

**Authors:** Julia Jensen-Kroll, Tobias Demetrowitsch, Ingrid Clawin-Rädecker, Martin Klempt, Silvio Waschina, Karin Schwarz

**Affiliations:** ^1^ Institute of Human Nutrition and Food Science, Division of Food Technology, Kiel University, Kiel, Germany; ^2^ Federal Research Institute of Nutrition and Food, Department of Safety and Quality of Milk and Fish Products, Max Rubner-Institute, Kiel, Germany; ^3^ Federal Research Institute of Nutrition and Food, Department of Microbiology and Biotechnology, Max Rubner-Institute, Kiel, Germany; ^4^ Institute of Human Nutrition and Food Science, Division of Nutriinformatics, Kiel University, Kiel, Germany

**Keywords:** caco-2 cells, oligosaccharides, GOS, MOS, metabolomics, FT-ICR-MS, pathway enrichment analysis, mass spectrometry

## Abstract

Milk oligosaccharides (MOS) and galactooligosaccharides (GOS) are associated with many benefits, including anti-microbial effects and immune-modulating properties. However, the cellular mechanisms of these are largely unknown. In this study, the effects of enriched GOS and MOS mixtures from caprine and bovine milk consisting mainly 6'-galactosyllactose, 3'-sialyllactose, and 6'-sialyllactose on Caco-2 cells were investigated, and the treatment-specific metabolomes were described. In the control, the cells were treated with a sugar mix consisting of one-third each of glucose, galactose and lactose.

A local metabolomics workflow with pathway enrichment was established, which specifically addresses DI-FT-ICR-MS analyses and includes adaptations in terms of measurement technology and sample matrices. By including quality parameters, especially the isotope pattern, we increased the precision of annotation. The independence from online tools, the fast adaptability to changes in databases, and the specific adjustment to the measurement technology and biomaterial used, proved to be a great advantage.

For the first time it was possible to find 71 active pathways in a Caco-2 cell experiment. These pathways were assigned to 12 main categories, with amino acid metabolism and carbohydrate metabolism being the most dominant categories in terms of the number of metabolites and metabolic pathways. Treatment of Caco-2 cells with high GOS and glucose contents resulted in significant effects on several metabolic pathways, whereas the MOS containing treatments resulted only for individual metabolites in significant changes. An effect based on bovine or caprine origin alone could not be observed. Thus, it was shown that MOS and GOS containing treatments can exert microbiome-independent effects on the metabolome of Caco-2 cells.

## 1 Introduction

Individual milk oligosaccharides (MOS) such as 3'-sialyllactose and 6'-sialyllactose, and galactooligosaccharides (GOS) such as 6'-galactosyllactose are attributed with numerous positive properties. These include prebiotic, anti-adhesive, anti-microbial, and immune-modulating effects (Bode, 2012). In addition, they have been shown to affect cell proliferation, differentiation, and maturation *in vitro* models (Holscher et al., 2014, 2017). The activity of oligosaccharides varies according to chain length, type of linkage, and degree of polymerization (Sanz et al., 2006; Boehm and Moro, 2008). MOS are found in all mammalian milks, but the milks differ in content, composition, and diversity of oligosaccharides (Kunz et al., 2000; Boehm and Stahl, 2007). Caprine milk has a higher level of sialylated oligosaccharides compared to bovine milk and a profile that is close to human milk in terms of total oligosaccharide content and the occurrence of fucosylated, sialylated, and neutral lactose-derived oligosaccharides (Albrecht et al., 2014; Oliveira et al., 2015). GOS, as an ingredient of infant formula, are primarily produced synthetically by enzymatic trans-glycosylation of lactose, usually using specific ß-galactosidases (Torres et al., 2010). Since the enzymes of the human body are only able to cleave the ß-glycosidic bond of lactose, ß-glycosidic linked oligosaccharides reach the large intestine undigested (Engfer et al., 2000). In the intestine they are primarily metabolized by the human intestinal microbiota. Lactobacilli and bifidobacteria are mainly involved in the fermentation of prebiotics (Macfarlane et al., 2008). However, MOS, which also include the sialyllactoses, are only fermented by bifidobacteria, while ß-GOS, to which galactosyllactose belongs, are fermented by both bifidobacteria and lactobacilli (Gänzle and Follador, 2012). The main fermentation products of prebiotics are short-chain fatty acids such as acetate, butyrate, and propionate, and the disaccharide lactate. Butyrate serves as main energy source for differentiated intestinal epithelial cells such as colonocytes, but has a negative effect on cell proliferation in undifferentiated cells. This effect is also referred as “butyrate paradox” (Salvi and Cowles, 2021). However, the oligosaccharides can also interact directly with intestinal cells and can cause microbiota-independent effects. This was also shown by the *in vitro* experiments on cell proliferation with various differentiated and undifferentiated cells as well as various oligosaccharides by Kuntz et al. (2008). However, metabolomic effects of oligosaccharides on differentiated cells are lacking.

A well-established *in vitro* system to study the interaction between intestinal cells and oligosaccharides are Caco-2 cells. Caco-2 cell lines are a continuous line of heterogeneous human epithelial colorectal adenocarcinoma cells (Fogh et al., 1977). In the differentiated and polarized state, they form monolayers with characteristic properties of enterocytes of the small intestine (Pinto et al., 1983). These include the formation of tight junctions, microvilli, enzymes such as disaccharidases and peptidases, and transport systems for sugars, vitamins, and amino acids. In addition to studies on transport mechanisms, Caco-2 cells have also been used to study the anti-inflammatory and immunomodulatory properties of various compounds due to their ability to produce cytokines such as IL-6, IL-8, and TNFα. Moreover, anticancerogenic and antioxidant effects have been studied in the Caco-2 model system (Iftikhar et al., 2020).

Previous studies on the Caco-2 metabolome regarding cell differentiation (Lee et al., 2009) or with other treatments for example gold nano particles (Gioria et al., 2016), crab waste (Ó Fearghail et al., 2021) or inulin (Lamers et al., 2003) primarily use nuclear magnetic resonance (NMR) or liquid chromatography-mass spectrometry (LC-MS) analyses. In addition, Ó Fearghail et al. (2021) used global overviews to derive metabolomic changes, Lee et al. (2009) considered a selected set of hydrophilic metabolites, and Gioria et al. (2016), and Lamers et al. (2003) restricted their analyses to the level of altered metabolites. A deeper insight into the Caco-2 metabolome is missing.

Direct injection mass spectrometry analyses (DI-MS), e.g., with Fourier-transform ion cyclotron resonance mass spectrometry (FT-ICR-MS), is able to cover a large range of metabolites in a short measurement time, even for complex sample matrices without prior chromatographic separation. In addition, it has extremely high resolution (xHR) in comparison to various LC-MS analytical techniques and very high sensitivity in comparison to NMR (Aharoni et al., 2002; Witting et al., 2015; Zhu et al., 2021). In particular, the high sensitivity may contribute to obtaining a deeper insight into the metabolome of Caco-2 cells.

The first aim of the study was to establish a metabolomics workflow which improves the identification level for DI-FT-ICR-MS analyses, which is usually referred to the lowest level G (Rampler et al., 2021) or 1 as defined by the Metabolomics Standards Initiative (Sumner et al., 2007; Creek et al., 2014), and to investigate the presence of isobaric compounds. The second aim was to characterize the Caco-2 metabolome in detail, objectively applying the new workflow and investigating, how enzymatically produced GOS compared with caprine and bovine MOS fractions influence the cell metabolome.

## 2 Experimental section

### 2.1 Materials

All solvents listed were obtained from Carl Roth, Karlsruhe, Germany. All consumables were purchased from Sarstedt, Nümbrecht, Germany.

#### 2.1.1 Oligosaccharides

Oligosaccharides were produced according to Frenzel et al. (2015) and Altmann et al. (2015; 2016). In brief, GOS was prepared using the enzyme HaLactase from *K. lactis*. The high GOS **(HG)** fraction contained different mono-, di-, tri- and tetrasaccharides, with 6'-galactosyllactose as main product. Bovine and caprine skimmed ultrafiltrated (UF) milks were subjected to nanofiltration. For better separation of MOS from residual sugars, lactose in the UF-permeate was hydrolyzed by ß-galactosidase. High GOS/bMOS **(GM)** from cow’s milk fraction contained the bovine MOS N-acetyl-β-D-galactosamine-lactose, 6'-sialyllactose, and 3'-sialyllactose as main products. Low GOS/cMOS **(LGM)** from goat’s milk contained 6'-sialyllactose and 3'-sialyllactose as main component of the caprine MOS fraction beside other minor MOS. Both fractions contained in addition comparable amounts of GOS, monosaccharides and lactose from the lactose hydrolysis during sample preparation and further milk components like milk salts. Control **(C)** consisted of glucose, galactose, and lactose (1/1/1, w/w/w), as they were also part of the GOS and MOS fractions. All GOS/MOS contents were determined using high-performance anion exchange chromatography with pulsed amperometric detection (HPAEC-PAD). A detailed overview of the oligosaccharides compositions is summarized in Table 1.

**TABLE 1:** Overview of the different oligosaccharide compositions.

	High GOS (HG)	High GOS/bMOS (GM)	Low GOS/cMOS (LGM)	Control (C)
GOS/MOS	69.1%	12%	3.3%	—
Glucose	1.2%	16.6%	15.2%	33.3%
Galactose	2.0%	12.5%	13.3%	33.3%
Lactose	27.7%	0.14%	—	33.3%

GOS, Galactooligosaccharides; MOS, Milk oligosaccharides; b, bovine; c, caprine

### 2.2 Methods

#### 2.2.1 Cell culture

Human colon adenocarcinoma cell line Caco-2 (Toni Lindl, München, Germany) between passage 24 and 50 were cultured in T-175 polystyrene flasks (Sarstedt, Nümbrecht, Germany) with Caco-2 medium (45% Dulbecco’s Modified Eagle Medium [DMEM], low glucose, 45% Ham’s F12, 9% fetal calf serum [FCS], 0.9% non-essential amino acids [all PAA Laboratories GmbH, Austria], and insulin [10 μg ml^−1^]; Biochrome AG, Berlin, Germany) at 37°C and 5% CO_2_. For all experiments cells were seeded at a density of 1 × 10^5^–2×10^5^ cells cm^−2^ to ensure confluency after O/N incubation. Medium was changed every second day. After 20 days, cells were treated in accordance with the experiments of Lafontaine et al. (2020) for 24 h with HG, GM, LGM, or (as a control) with a sugar mixture (galactose, glucose and lactose [1/1/1, w/w/w]) and at a concentration of 25 mg ml^−1^ (Zenhom et al., 2011). After this incubation, cells were visually inspected, medium was removed, cells were washed twice with ice-cold deionized H_2_O, snap frozen in liquid nitrogen to quench cell metabolism, and stored at −80°C. 8 replicates were made for each treatment.

#### 2.2.2 Sample preparation

Cells were removed from the culture flasks under standardized conditions using a cell scraper. Thereafter, 3 times 2 ml of ultra-pure water with 0.1% (v/v) formic acid was added and treated for 2 min each. Cell disruption was conducted by ultrasonication (HD 2070 Bandelin electronics, Berlin Germany; 40% in seven cycles for 5 min, using a MS 73 rod) on ice. The cell lysate was dried by means of a speedvac (VR-maxi St. a-1/CT 60E, Heto-Holten A/s, Allerd, Denmark) and resuspended in 1.5 ml ultra-pure water. Extraction was performed using a modified SIMPLEX approach based on Matyash et al. (2008). 500 µL methanol and 4 ml methyl tert-butyl ether were added to 500 µL of sample and vortexed. Samples were incubated at room temperature for 30 min in an overhead shaker at 25 rounds per minute (Trayster, Ika, Staufen, Germany). 500 µL ultra-pure water was added, followed by incubation for 10 min at room temperature (without shaking). Finally, all samples were centrifuged for 5 min at 1,000 *g* (Allegra X-30R/SX 4400, Beckman Coulter, Krefeld, Germany). The upper phase was transferred to a new tube and kept at 4°C. A second extraction cycle was conducted as described above. The resulting upper lipophilic phase and middle hydrophilic phase were transferred into 15 ml tubes, and the lower proteinogenic phase was discarded. The phases were dried with a speedvac and resuspended with the following liquids: The hydrophilic compounds (middle phase) were resuspended in methanol/water (50/50, v/v) with 0.1% acetic acid (500 µL). The lipophilic compounds (upper phase) were resuspended in isopropanol/chloroform (3/1, v/v) with 0.1% acetic acid (500 µL). Samples were stored at −80°C until measurement. This work focused on the investigation of the hydrophilic compounds.

#### 2.2.3 Workflow for the metabolomics approach

**FIGURE 1:**
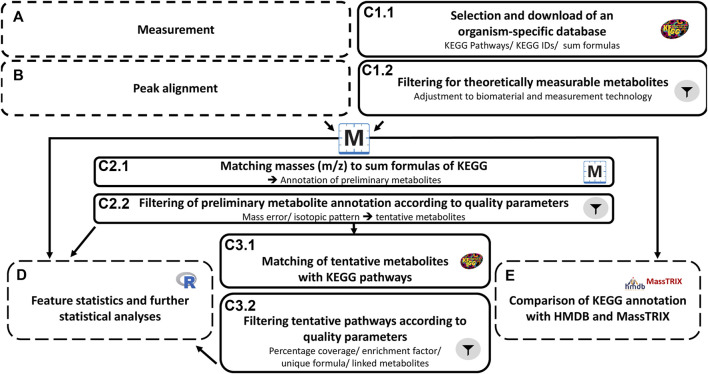
Workflow metabolomics approach. The metabolomics workflow was divided into five main steps **(A–E)**. Samples were measured **(A)** and signals were aligned *via* instrument-specific software **(B)**. The data analysis **(C)** was further divided into six steps. This included the selection and download of an organism-specific database, metabolite annotation and pathway mapping, and in addition, specific filter elements with adjustments regarding biomaterial and measurement technique (C.1.2/C2.2/C3.2) to reduce false-positive results. In parallel with metabolite annotation, feature statistics were performed **(D)** based on exported bucket tables after step B. These data were also used for comparative analyses in web databases **(E)**.

The workflow for the metabolomics analyses is presented in Figure 1. After **measurement** *via* FT-ICR-MS (A), **peak alignment** (B) was conducted using MetaboScape 4.0 SR1 (Bruker, Germany). For **data analysis** and metabolite annotation (C) in the first step (C1.1), an organism-specific database was selected and downloaded (KEGG *hsa* pathways with corresponding KEGG IDs and sum formulas; (Kanehisa, 2000)). The resulting KEGG IDs and the corresponding formulas were combined to form an annotation list, in which isobaric compounds were grouped. In the following step (C1.2), the entries were filtered considering the specific biomaterial and the measurement technique. Only those compounds that are assigned a sum formula in KEGG were considered. Formulas with undefined moieties (e.g., “R” and “X”) and n-fold repetitions of parts of the sum formulas [e.g., “(C_12_H_20_O_11_)_n_”] were not considered for further analysis. In addition, only sufficiently large (≥60Da) and thus potentially measurable compounds were considered. Drugs and associated pathways were also excluded. The resulting, cleaned local database was then used for metabolite annotation in MetaboScape. Thereafter, the theoretical masses were calculated based on the sum formulas and compared and matched with the measured masses (C2.1). The resulting preliminary annotations were again subjected to filtering according to quality parameters (C2.2). These quality parameters included the mass error and the isotope pattern (see 2.2.5). Confirmed formulas and thus confirmed IDs of tentative metabolites were merged into a new export file, which in turn was used to map KEGG compounds to pathways in which the compounds participate (C3.1). The resulting pathway information and associated metabolites were used for pathway enrichment analysis. To evaluate and filter the enrichments, the following quality parameters were considered (C3.2): percentage of coverage, enrichment factor, occurrence of pathway-specific sum formulas, and number of linked metabolites (see 2.2.7).

(D) In parallel, non-targeted **feature statistics** were performed and included principal component analysis (PCA) as well as differential feature statistics to identify differences in feature intensities between treatments. In the final step (E), the results of the workflow were compared with two different web-databases.

For the **comparisons** MassTRIX (Version 3.0, Status 06/21, Helmholz Center, Munich, Germany; (Suhre and Schmitt-Kopplin, 2008)) and HMDB (Version 4.0, Status 06/21, University of Alberta, Canada; (Wishart et al., 2018)) were used with the following parameters: mass error <2 ppm, tolerated ions [M + H]^+^, [M + Na]^+^, [M + K]^+^, [M-H]^−^, and [M + Cl]^−^. Only adducts, which add elements to the molecular formula that are not carbon-based or based on neutral loss (such as the loss of H_2_O or carbon dioxide) were used. Further settings for MassTRIX: database KEGG, HMDB, Lipidmaps without isotopes, organism *Homo sapiens*. For the comparative web-annotation, the data resulting from Step B were used. The measured masses were exported as a bucket table (including all possible adducts). From the web-based comparisons, only metabolites for which a KEGG ID was available were used for further analyses. Based on this data, a new local database was created for MetaboScape, which also passed through workflow steps C1.1 to C3.1.

#### 2.2.4 FT-ICR-MS measurements

For the mass spectrometric analysis, a FT-ICR-MS system (7T, SolariXR, Bruker, Bremen, Germany) equipped with an ESI source was used. For the direct injection (DI), an Infinity 1260 HPLC autosampler and pump (Agilent, Waldbronn, Germany) was linked. The eluent consisted of methanol and water (50:50, v/v) with 0.1% acetic acid. For analyses samples were ionized in positive or negative modes by electro spray ionization. The mass range was between 60 and 1,500 m/z. The main parameters were: Nitrogen as dry gas (4 L/min and 200°C) and nitrogen as nebulizer (1 bar); the time-of-flight section was set to 0.35 ms; the quadrupole mass was set to 200 m/z; the quadrupole RF was 2 MHz and the sweep excitation power of the ICR-cell was 18%. All methods were calibrated using sodium trifluoroacetate. The stability of the measurement was monitored using quality control samples (QC). The QC represents a pool of all samples measured every 20th analysis (Demetrowitsch et al., 2015).

#### 2.2.5 Data handling and metabolite annotation

FT-ICR-MS data were processed by MetaboScape 4.0 (Bruker, Bremen, Germany). All datasets were recalibrated by matrix compounds within the samples (e.g., glucose and amino acids) with a tolerated error <0.5 Da. To reduce noise, only peaks were selected which were present in at least 20 of 96 samples. Further, the intensity threshold was set to 500,000 counts.

For metabolite annotation, the database resulting from the workflow was used with adaptations to the organism, i.e., *hsa*, and to the measurement technique. Peaks were annotated if the mass error was below 2 ppm and if the mSigma (isotope error) was below 300 (quality parameters). The annotation was considered individually for each ion: [M + H]^+^, [M + Na]^+^, and [M + K]^+^ (positive mode), and [M-H]^−^ and [M + Cl]^−^ (negative mode), respectively. The annotated data were exported as tab-separated tables of the feature intensities for subsequent statistical analysis in R version 4.1.1. If a metabolite was present in the form of several ions, the ion with the highest intensity count was used for the statistical analysis.

#### 2.2.6 Multivariate and differential feature statistics

The feature signal intensity tables that were exported from MetaboScape were read into R version 4.1.1 for further analysis (R Core Team, 2021). First, the feature intensities within each sample were normalized by dividing each value by the median intensity value (i.e., median normalization, Välikangas et al., 2018). Second, normalized intensity values were log2-transformed. Taken together, the formula for normalization and transformation was:

$${\tilde{x}}_{ij}=\log_{\;2}\left(\frac{x_{ij}}{med\left(x_{i}\right)}\right)$$ (1)

Where $x_{ij}$ denotes the intensity value of feature j in sample i and $med\left(x_{i}\right)$ the median feature intensity in sample i . Third, the normalized and transformed values ( ${\tilde{x}}_{ij}$ ) were subjected to pareto-scaling per feature using the formula:

$${\overset{˘}{x}}_{ij}=\frac{{\tilde{x}}_{ij}-mean\left({\tilde{x}}_{j}\right)}{\sqrt{sd\left({\tilde{x}}_{j}\right)}}$$ (2)

Where $mean\left({\tilde{x}}_{j}\right)$ denotes the mean normalized and transformed intensity of feature j and $sd\left({\tilde{x}}_{j}\right)$ the standard deviation of ${\tilde{x}}_{j}$ . All feature values below the limit of detection (LOD) were considered as missing values (i.e., NA) for the described normalization, transformation, and scaling. Following the pareto-scaling, missing values for features, which were missing in less than 25% across all samples, were imputed using the k-nearest neighbor algorithm (with k = 10) as implemented in the function “knn.impute” from the R-package “bnstruct” version 1.0.11 (Franzin et al., 2017). Remaining missing values were imputed with the minimum feature intensity across all features and samples. PCA was performed on pre-processed and imputed feature intensities using the R-function “prcomp”. Differential analysis of individual feature intensities between treatment groups were performed using linear mixed effect models in the R-package “lme4” version 1.1-27.1 (Bates et al., 2014). Treatment type was defined as a fixed effect, while the intercept of biological sample ID was defined as a random effect. *p*-values that describe the significance of different feature intensities between treatments were obtained by likelihood-ratio tests of the full model (with treatment as a fixed effect) against the reduced model without treatment type as a variability-explaining factor. Median fold changes (Hoffmann et al., 2002) of feature intensities were calculated to estimate differential metabolite concentrations between two different treatments.

#### 2.2.7 Pathway enrichment analysis

Pathway enrichment analysis was performed to address two questions: 1) How many metabolites of a pathway were found in the metabolomics data relative to the number of metabolites in the same pathway that could theoretically be measured using the FT-ICR-analysis if above the LOD? 2) Which pathways are enriched in metabolites that have been identified to be significantly abundant, dependent on the treatment type based on pairwise-treatment comparisons using a linear mixed effect model statistic (see 2.2.6)? The metabolites ATP, ADP, AMP, NAD, NADH, NADP, NADPH, coenzyme A, phosphate, and diphosphate were not considered in the pathway enrichment analysis as these metabolites participate in a wide range of diverse pathways and are therefore not informative for the activity of specific pathways. Furthermore, only metabolites that are theoretically measurable by the FT-ICR-MS approach were included in the pathway enrichment analysis, which excludes metabolites with a mass of less than 60 Da and metabolites with ambiguous sum formula (e.g., with “R” indicated undefined moieties). Enrichment factors of significant treatment-dependent metabolites for each pathway *i* were calculated using the formula

$$a_{i}=\frac{n_{sig,i}/n_{anno,i}}{n_{sig}/n_{anno}},$$ (3)

Where $n_{sig,i}$ is the number of significant metabolites in pathway i, $n_{sig}$ the number of significant metabolites across all pathways, $n_{anno,i}$ the number of annotated metabolites in pathway i, and $n_{anno}$ the number of all annotated metabolites across all pathways. The enrichment factors of the pathways based on annotated metabolites relative to all theoretically measurable metabolites, using the FT-ICR-MS, were calculated by

$$a_{i}=\frac{n_{anno,i}/n_{meas,i}}{n_{anno}/n_{meas}},$$ (4)

where $n_{meas,i}$ corresponds to the number of measurable metabolites in pathway i and $n_{meas}$ the number of all measurable metabolites across all pathways. Significance levels of pathway enrichments were obtained using the Fisher’s Exact Test for Count Data. The calculation of the enrichment factors was based strictly on sum formulas, with each sum formula being counted only once per pathway. For the enrichment analyses, the following quality parameters were considered: Percent coverage (>33.33%), occurrence of pathway characteristic sum formulas, and the number of linked non-isobaric metabolites per pathway (min. 3). Only pathways that fulfilled at least one of these criteria were considered in subsequent analyses.

## 3 Results

In a semi-targeted metabolomics approach, the bovine and caprine milk- (b- and cMOS) and galactooligosaccharide (GOS) treatment-specific Caco-2 metabolomes should be investigated. For this purpose, four different sugar combinations were used: a high content of GOS **(HG)**, a combination with a high content of GOS/bMOS **(GM)**, a combination with a low GOS/cMOS content **(LGM)**, and a control **(C)** which consisted of one-third each of glucose, galactose, and lactose. Since the treatments were not pure substances, and also contained e.g., mono- and disaccharides, the different treatments were compared in six combinations.

• Each individual treatment vs. control
• The two GOS/MOS treatments against each other
• The two GOS/MOS treatments individually against the high GOS treatment

The multi-comparisons allow us to distinguish whether differences between metabolomics profiles are due to the monosaccharides, the disaccharides, or the presence of GOS or MOS. Moreover, general and concentration-dependent effects could be inferred (e.g., due to different amounts of glucose and galactose in the four different treatments).

### 3.1 Results of the metabolomics workflow

A local workflow has been developed and applied, which specifically addresses the highly complex metabolomics data from DI-FT-ICR-MS analyses. This workflow includes five basic elements: Measurement **(A)**, peak alignment **(B)**, data analysis **(C)**, and statistics **(D)**. Element **E** evaluates comparability with workflows using web-based approaches with databases or online tools. A key objective of the presented workflow is the reduction of false-positive results at the level of metabolites and pathways through specific filtering steps using various quality parameters (see 2.2.5/2.2.7).

Based on this workflow we identified all metabolites and associated pathways characterizing the Caco-2 metabolome and treatment-specific effects using different sugar mixtures. The numeric results of the individual steps of the metabolomics workflow are shown in Table 2.

**TABLE 2:** Overview of results of the workflow for the metabolomics approach; subdivided according to steps and their respective output.

Step		Output
**A/B**	**Measurement** and **peak alignment** (20 of 96 samples)	5,945 features (2,949 pos. ionized/2,996 neg. ionized)
**C**	**Data analysis**	
C1.1	Download KEGG pathways and related metabolites	2077 sum formulas/3177 KEGG IDs
C1.2	Filtering for theoretical measurable metabolites	1,665 sum formulas/2603 KEGG IDs
C2.1/C2.2	Matching *via* MetaboScape and filtering according to quality parameters^1^	211 sum formulas/468 KEGG IDs
C3.1	Matching metabolites to KEGG pathways, ≥3 Hits	83 pathways (*hsa*) without global overviews
C3.2	Filtering pathways according to quality parameters^2^	71 pathways/202 sum formulas/445 KEGG IDs
**D**	**Statistics**	
	Significant features	1700 features (437 pos. ionized/1,263 neg. ionized)
	Significant metabolites	103 significant sum formulas/275 significant KEGG IDs
	Significant pathways	25 significant pathways
**E**	**Comparisons**	
	HMDB	Total hits 1,372 sum formulas/9,448 IDs
	KEGG related hits	397 sum formulas/735 KEGG IDs
	Validation *via* MetaboScape, re-matching	360 sum formulas/679 KEGG IDs
	
	MassTRIX	Total hits 948 sum formulas/4,296 IDs
	KEGG related hits	766 sum formulas/1857 KEGG IDs
	Validation *via* MetaboScape, re-matching	642 sum formulas/1634 KEGG IDs

^1^ Quality parameters: mass error, isotopic pattern, see 2.2.5.

^2^ Quality parameters: percentage coverage, unique sum formulas, min. 3 linked non-isobaric metabolites, see 2.2.7.

Peak alignment resulted in 5,945 features (B), a feature is defined as the measured mass (m/z) without any annotation. Based on the annotation list obtained after the first filter step (C.1.2), containing all potentially measurable metabolites and sum formulas, 211 sum formulas with 468 corresponding KEGG IDS were assigned to the 5,945 features. This corresponds to a percentage share of 3.6%. Matching of metabolites to the corresponding metabolic pathways by KEGG mapping resulted in 83 preliminary metabolic pathways with at least 3 hits (C3.1); metabolites listed in 2.2.7 were excluded. Based on the specified quality parameters of the pathway enrichment analyses, a further 12 of 83 pathways were excluded (C3.2). Multiple comparisons in the statistical analyses revealed that 25 of the remaining 71 metabolic pathways were significantly regulated (D). Step E shows the difference between annotation with web-based approaches and the results validated *via* MetaboScape considering the quality parameters.

### 3.2 Pathway enrichment analyses

Pathway enrichment analyses were used to reduce the number of false-positive annotations by applying various quality criteria (see 2.2.5/2.2.7). These included a pathway coverage of at least 33.33%, the occurrence of at least three directly linked non-isobaric metabolites within one pathway, and the occurrence of unique sum formulas.

Pathway mapping based on the annotated metabolites resulted in 83 preliminary pathways having a minimum of 3 hits. Based on all sum formulas that could potentially be determined *versus* those sum formulas which have been determined, a coverage rate of 22.86% is expected across all 83 pathways. 46 pathways were above the expected value, eight of them significantly (*p*-value < 0.05). Applying the quality parameters resulted in 35 pathways passing the 33.33% level, 42 containing at least one unique molecular formula and, 28 having at least 3 directly linked metabolites. In the following the percentage distribution of the 83 pathways based on the number of fulfilled criteria is listed:

• 5 of 83 pathways fulfilled 3 criteria, corresponding to 6.02%
• 24 of 83 pathways fulfilled 2 criteria, corresponding to 28.92%
• 42 of 83 pathways fulfilled 1 criterion, corresponding to 50.60%
• 12 of 83 pathways did not fit any of the criteria, corresponding to 14.46%

12 pathways did not meet any of the criteria and were thus excluded from further analyses. For detailed information see Supplementary Material S1

### 3.3 The treatment specific metabolome of the Caco-2 cells is characterized by 12 pathway categories

**FIGURE 2:**
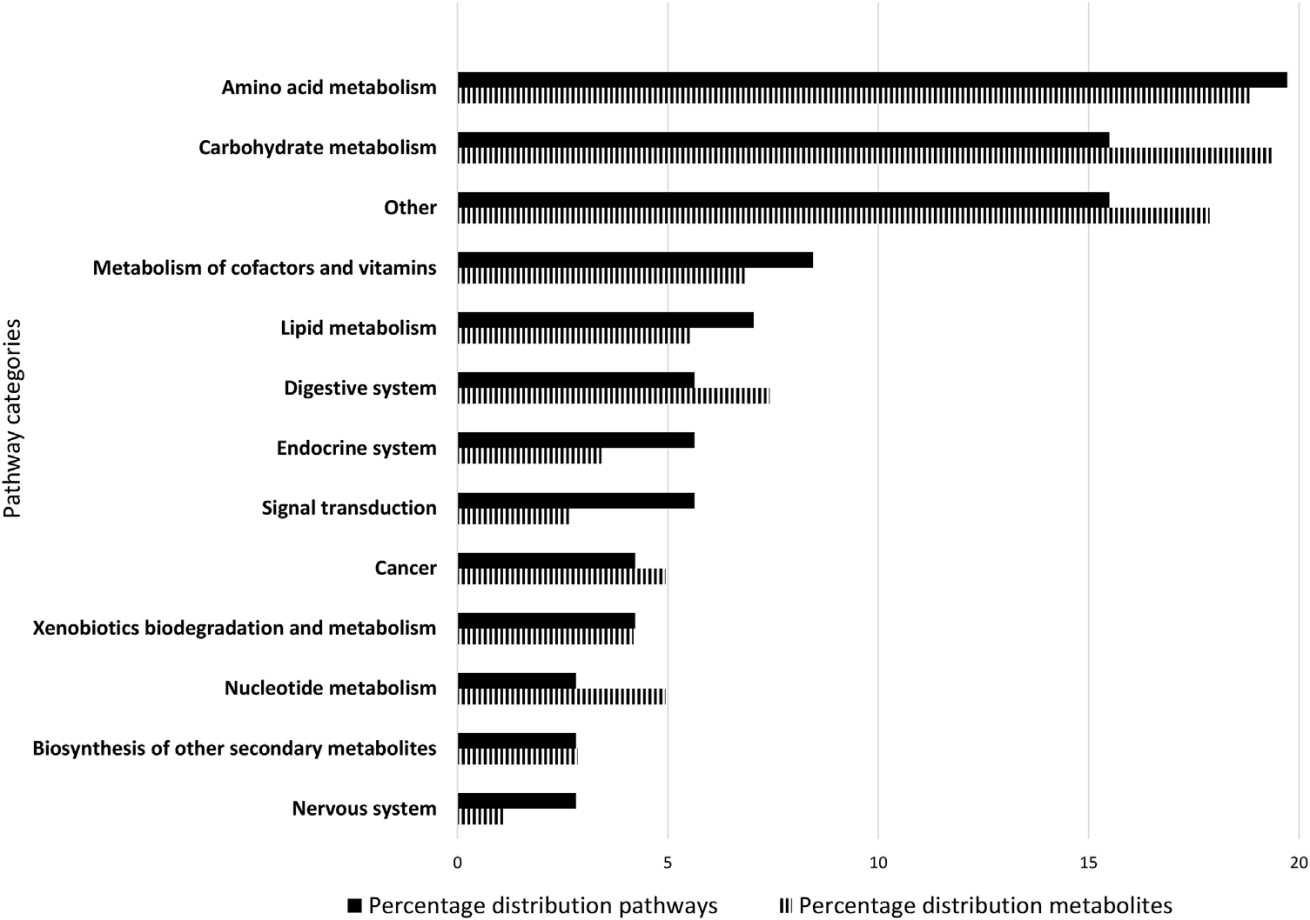
Composition of the Caco-2 metabolome. The 71 metabolic pathways of the Caco-2 metabolome were classified into 13 categories. The classification was based on the KEGG nomenclature. Main categories to which only one pathway belongs were aggregated under “Other”. The bar chart shows the percentage of metabolic pathways assigned to main categories in black, and the corresponding distribution of metabolites in striped. The figure is ranked according to the percentage of pathways assigned from largest to smallest share.

All 71 remaining identified pathways, which fulfilled at least one criterion, were assigned to 24 different main categories based on the KEGG nomenclature (For detailed information see Supplementary Material S1). 12 main categories included more than one pathway. Main categories including only one pathway were aggregated under “Other” (Figure 2). More metabolites were attributable to carbohydrate metabolism (19.39%) and amino acid metabolism (18.82%) than other categories.

### 3.4 Hub metabolites

Metabolites that occurred in the most of the 71 pathways, so called hub metabolites, are listed in Table 3. Glucose followed by its phosphorylated form glucose-6-phosphate and glutamate occurred in 21, 16 and 17 pathways, respectively. In some cases, the hub metabolites were also significantly regulated in the different comparisons.

**TABLE 3:** Overview of the most commonly found hub metabolites in the pathway enrichment analyses, with p-values of the different comparisons; ranked by the number of occurrences in pathways, superscripts indicate significant upregulation in the corresponding treatments, epimers are summed up, “xxx” no significant differences in this comparison.

KEGG ID	Metabolite name	Number of pathways	HG vs. C	HG vs. GM	HG vs. LGM	LGM vs. C	GM vs. C
			*p*-value	*p*-value	*p*-value	*p*-value	*p*-value
C00031	D-glucose*	21	7.42E-05^C^	0.01685^GM^	0.00091^LGM^	xxx	xxx
C00267	alpha-D-glucose*						
C00221	beta-D-glucose*						
C00025	L-glutamate	17	2.24E-06^HG^	2.42E-05^HG^	0.00086^HG^	xxx	xxx
C00092	D-glucose 6-phosphate**	16	0.00082^C^	0.00196^GM^	0.00196^LGM^	xxx	xxx
C00668	alpha-D-glucose-6-phosphate**						
C01172	beta-D-glucose-6-phosphate**						
C00064	L-glutamine	13	6.72E-06^HG^	0.00106^HG^	0.001227^HG^	xxx	xxx
C00049	L-aspartate	11	9.91E-06^HG^	6.57E-07^HG^	1.01E-05^HG^	xxx	xxx
C01245	D-myo-inositol 1,4,5-trisphosphate	11	xxx	0.01313^GM^	xxx	xxx	0.03947^GM^
C00085	D-fructose 6-phosphat**	11	0.00082^C^	0.00196^GM^	0.00196^LGM^	xxx	xxx
C05345	beta-D-fructose 6-phosphate**						
C00082	L-tyrosine	10	xxx	xxx	xxx	xxx	xxx
C00065	L-serine	9	0.03272^HG^	0.01103^HG^	xxx	xxx	xxx
C00095	D-fructose*	9	7.42E-05^C^	0.01685^GM^	0.00091^LGM^	xxx	xxx
C02336	beta-D-fructose*						
C00029	UDP-glucose	8	xxx	xxx	xxx	xxx	xxx
C00078	L-tryptophan	7	xxx	xxx	xxx	xxx	xxx
C00062	L-arginine	7	xxx	xxx	xxx	xxx	xxx
C00079	L-phenylalanine	7	xxx	xxx	xxx	xxx	xxx
C00103	D-glucose 1-phosphate**	7	0.00082^C^	0.00196^GM^	0.00196^LGM^	xxx	xxx
C00183	L-valine	7	xxx	xxx	xxx	0.04031^LGM^	0.03947^GM^
C00188	L-threonine	7	0.00010^HG^	0.00014^HG^	0.00044^HG^	xxx	xxx
C00148	L-proline	6	xxx	xxx	xxx	xxx	xxx
C00186	L-lactate	6	xxx	xxx	xxx	xxx	xxx
			**10(5/5)** ^***^	**11(5/6)** ^***^	**9(4/5)** ^***^	**1(1/0)** ^***^	**2(2/0)** ^***^

*Isobaric compounds based on C6H12O6.

**Isobaric compounds based on C6H13O9P.

***Total number of significant metabolites (number of metabolites in treatment/number of metabolites in treatment).

### 3.5 Presence of isobaric compounds

For several cases, it is possible to demonstrate the presence, i.e., co-existence of various isobaric metabolites, or to exclude the presence of individual isobars, if precursor or subsequent metabolites are present or not in the pathway.

In the case of fructose-6-phosphate and glucose-6-phosphate, it is possible to demonstrate the presence of both metabolites by using the pentose phosphate metabolism. This pathway fulfills all quality parameters of the pathway enrichment analyses (see 2.2.7), i.e., a percentage coverage of 48.83%, two metabolites with unique sum formulas, and more than 3 linked metabolites. Thus, the pathway is considered to be confirmed. Both metabolites fructose-6-phosphate and glucose-6-phosphate are present in this pathway and directly linked to each other, i.e., fructose-6-phosphate is derived from glucose-6-phosphate, and subsequent metabolites such as fructose-1,6-bisphosphate, sedoheptulose-7-phosphate, and 2-deoxy-ribose phosphates have also been detected. Therefore, the co-occurrence of both isobars is plausible.

Furthermore, based on ascorbate and aldarate metabolism, it was possible to exclude the arabinonates, since neither precursor molecules nor subsequent metabolites were detected. But the presence of isobaric compounds xylonates and lyxonates is proposed, since two precursor molecules were detected. Ascorbate and aldarate metabolism fulfilled all 3 criteria with a coverage rate of 42.31%

Based on pyrimidine metabolism, the presence of the isobars glutamine and 3-ureidoisobutyrate would be excluded, as they appear without directly linked metabolites in the pathway. However, glutamine can be confirmed by alanine, aspartate, and glutamate metabolism, as it is directly linked to two further metabolites in this pathway. Pyrimidine metabolism is (with 24.56%) below the minimum percentage coverage but fulfills the other two criteria. Alanine, aspartate, and glutamate metabolism fulfills only the minimum criterion of linked non-isobaric metabolites.

### 3.6 Treatment specific changes in the cell metabolome

**FIGURE 3:**
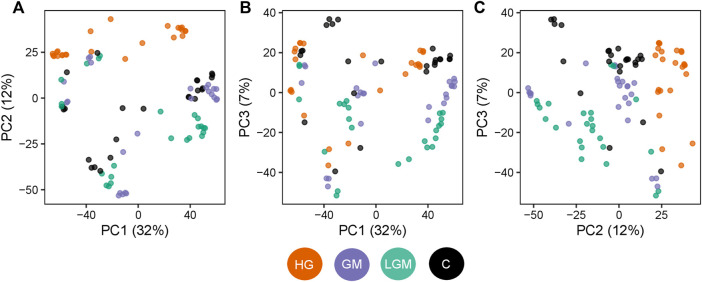
Treatment-specific changes in the cell metabolome. Scatterplot of the PCA scores for the three main principal components. The three collectively explain 51% of the variation observed in the metabolome profiles. Each point represents one sample and is colored based on the treatment type. Orange: samples with the high GOS content (HG), violet: samples with low GOS/MOS (LGM) content, green: samples with high GOS/MOS (GM) content, black: control. **(A)** PC1 vs. PC 2, **(B)** PC1 vs. PC3, **(C)** PC2 vs. PC3.

**FIGURE 4:**
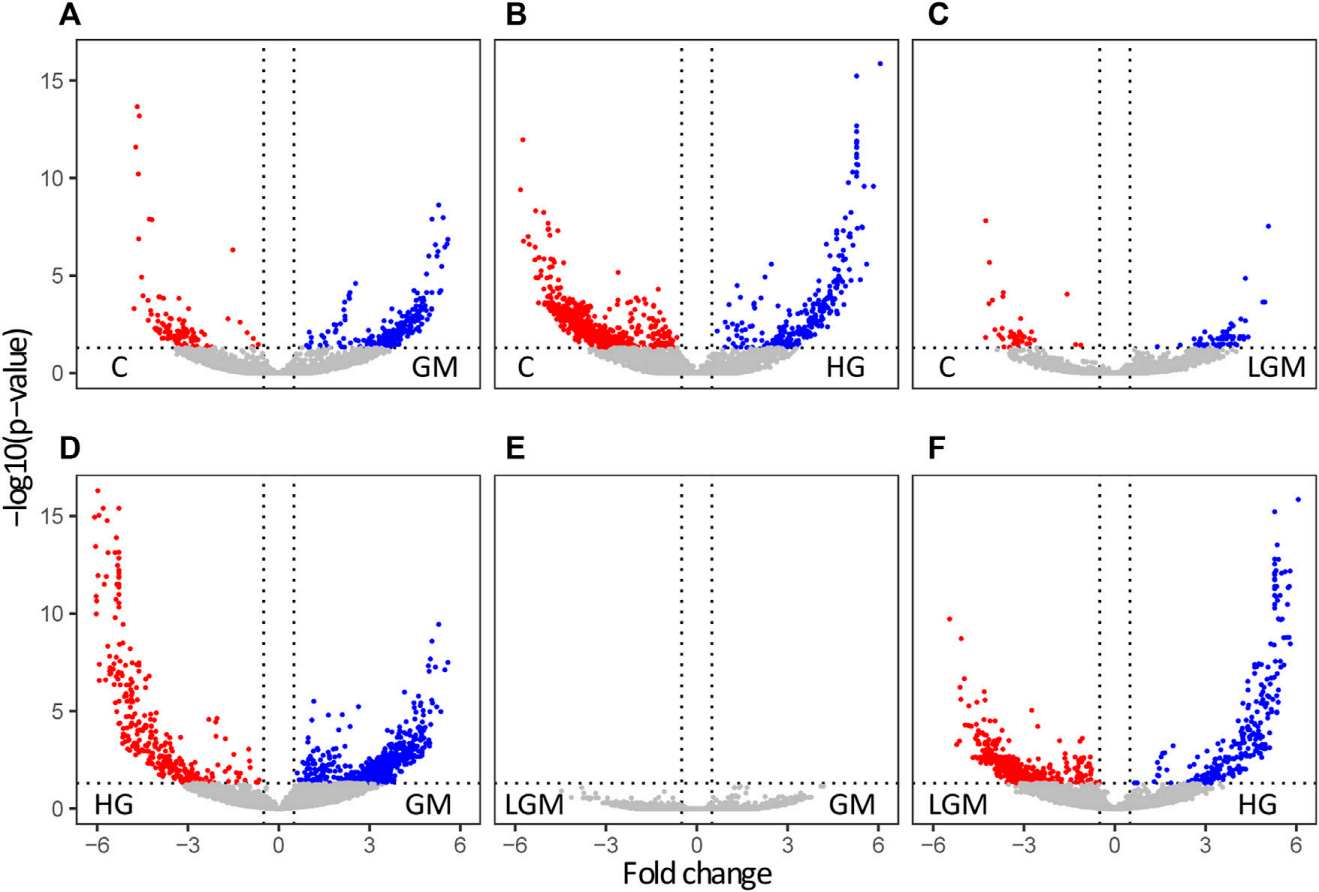
Treatment based significantly regulated features. Volcano plots of the significant features in pairwise comparisons of the different treatments. The data are plotted as −log10 (*p*-value) against the log2-fold change. Significantly regulated metabolites are highlighted in red and blue. Panels **(A–C)** show the significantly regulated features of the comparisons GM/HG/LGM vs. C, panel **(D)** the comparison of GM vs. HG, **(E)** the comparison of GM vs. LGM, and panel **(F)** HG vs. LGM.

To visualize overall similarities and differences in the cell metabolome caused by the treatments, and to give a general overview, a PCA was used. The metabolomes of the control, GM, and LGM exhibited similar scattering patterns in the PCA, whereas HG showed clear clustering in PC1 vs. PC2 and PC2 vs. PC3 in the score plots (Figure 3). In accordance with the PCA analyses most significant differences were found for treatments vs. control and for HG vs. other oligosaccharide treatments. There were no significant differences between MOS fractions of bovine and caprine origin (Figure 4).

### 3.7 Galactooligosaccharides and glucose led to significant changes at the pathway level

Across all previously described comparisons, there were a total of 25 significantly regulated pathways (*p* -value < 0.05). These pathways are categorized in Table 4 by their pattern of regulation. The metabolites significantly regulated within the pathways are listed in detail in the supplemental. Significantly regulated pathways were primarily found in comparisons of control and oligosaccharides treatments vs. high GOS level (HG). Across all comparisons, 13 of 25 pathways (nos. 1–13 in Table 4) can be clearly attributed to the effect of HG. Furthermore, there were 9 pathways (nos. 1–9 in Table 4) that showed differentiation when comparing different GOS levels, i.e., HG vs. GM and HG vs. LGM. Two pathways were attributed to the effect of glucose level, as they were upregulated in control vs. HG and GM vs. HG (nos. 14 and 15). In addition, 10 pathways (nos. 16-25) were found with no clear pattern of regulation and therefore are categorized as ambiguous effects. Effects based on the MOS containing sugar mixtures could not be clearly observed at pathway level.

**TABLE 4:** Significantly regulated pathways based on the various comparisons with p-values and regulation pattern; superscripts indicate significant upregulation of the pathway in the corresponding treatment.

No.	Pathway	HG vs. C	HG vs. GM	HG vs. LGM	LGM vs. C	GM vs. C
		*p*-value	*p*-value	*p*-value	*p*-value	*p*-value
	**Effect of high GOS**					
1	ABC transporters	0.0053^HG^	0.0115^HG^	0.0216^HG^	xxx	xxx
2	Alanine, aspartate and glutamate metabolism	0.0242^HG^	0.0213^HG^	0.0051^HG^	xxx	xxx
3	Aminoacyl-tRNA biosynthesis	0.0114^HG^	0.0020^HG^	0.0055^HG^	xxx	xxx
4	Arginine biosynthesis	0.0448^HG^	0.0406^HG^	0.0135^HG^	xxx	xxx
5	D-Glutamine and D-glutamate metabolism	0.0060^HG^	0.0054^HG^	0.0016^HG^	xxx	xxx
6	Histidine metabolism	0.0048^HG^	0.0041^HG^	0.0009^HG^	xxx	xxx
7	Porphyrin and chlorophyll metabolism	0.0448^HG^	0.0041^HG^	0.0135^HG^	xxx	xxx
8	Vitamin B6 metabolism	0.0448^HG^	0.0406^HG^	0.0135^HG^	xxx	xxx
9	Protein digestion and absorption	0.0171^HG^	0.0141^HG^	0.0079^HG^	xxx	xxx
	**Effect of GOS**					
10	Glycerophospholipid metabolism	0.0031^HG^	xxx	xxx	xxx	0.0179^GM^
11	Proximal tubule bicarbonate reclamation	0.0208^HG^	xxx	xxx	xxx	xxx
12	Taste transduction	0.0227^HG^	xxx	xxx	xxx	xxx
13	Central carbon metabolism in cancer	0.0335^HG^	xxx	xxx	xxx	xxx
	**Effect of Glucose**					
14	Fructose and mannose metabolism	0.0059^C^	xxx	xxx	xxx	xxx
15	Pentose phosphate pathway	0.0495^C^	xxx	0.0428^LGM^	xxx	xxx
	**Ambiguous Effect**					
16	Ascorbate and aldarate metabolism	xxx	xxx	0.0428^LGM^	xxx	xxx
17	Caffeine metabolism	xxx	0.0037^GM^	0.0186^LGM^	xxx	xxx
18	Cysteine and methionine metabolism	xxx	0.0019^HG^	0.0091^HG^	xxx	xxx
19	Glycerolipid metabolism	xxx	xxx	xxx	0.0372^LGM^	xxx
20	Glycine, serine, and threonine metabolism	xxx	0.0060^HG^	xxx	xxx	xxx
21	Insulin secretion	xxx	0.0345^GM^	xxx	xxx	xxx
22	Sulfur metabolism	0.0121^HG^	0.0107^HG^	xxx	xxx	xxx
23	Glutamatergic synapse	xxx	xxx	0.0377^HG^	xxx	0.0213^GM^
24	Purine metabolism	xxx	xxx	0.0436^HG^	xxx	xxx
25	Valine, leucine, and isoleucine biosynthesis	xxx	xxx	0.0377^HG^	xxx	xxx
		**16 (14/2)** ^*^	**14 (12/2)** ^*^	**16 (13/3)** ^*^	**1 (1/0)** ^*^	**2 (2/0)** ^*^

^*^Total number of regulated pathways (number of pathways in treatment/ number of pathways in treatment).

### 3.8 Further significantly regulated metabolites

A total of 275 metabolites were significantly regulated. Significant regulations of whole pathways could only be clearly attributed to GOS and glucose. In the following, metabolites that were not assigned to the significantly regulated pathways related to GOS and glucose effects are summarized. There were 68, 34, 23, and 6 metabolites caused by glucose, GOS, MOS fraction, and lactose effects, respectively. Based on the unique, non-isobaric sum formula, histidine, threonate, and glycerol-3-phosphate were identified as significantly regulated by GOS (HG/GM/LGM vs. C). Regulation of 2-isopropylmaleate, 1-aminopropan-2-ol-O-phosphate, and 2-amino-3-phosphonopropanoate was found for a high GOS amount (HG vs. C/GM/LGM) and of 3-dehydro-threonate for HG vs. C. Creatine was significantly regulated in the context of the MOS fraction (GM/LGM vs. HG and GM vs. C) and regulation of shikimate-3-phosphate, 3-hydroxy-2-methylpyridine-4,5-dicarboxylate, ribosyl-homocysteine, and 1-myo-inositol-1,4,5 triphosphate was identified for GM (GM vs. HG/C).

## 4 Discussion

The first aim of the study was to establish a local metabolomics workflow which improves the identification level for DI-FT-ICR-MS analyses, and to investigate the presence of isobaric compounds.

The second aim was to characterize the Caco-2 metabolome in detail, objectively applying the new workflow and investigating how milk oligosaccharide (MOS) and galactooligosaccharide (GOS) containing sugar mixtures influence the metabolome of Caco-2 cells.

### 4.1 Metabolomics workflow

Using the presented workflow, we were able to identify 71 pathways, 202 sum formulas, and 445 KEGG IDs based on 5,945 features measured. Comparing these numbers with the web-based annotations using HMDB (397 sum formulas and 735 KEEG IDs) or the web-based tool MassTRIX (766 sum formulas and 1857 KEGG IDs) the number of identified metabolites were lower in our workflow. The differences can be explained by our filtering steps, which included quality parameters that evaluated isotopic patterns and fine structures (see 2.2.5), thus leading to a higher precision in identification. Further, pathway enrichment analyses were used as filtering elements which resulted in a lower number of confirmed annotations. Further reduction was caused by the exclusion of drug metabolism pathways, and metabolites with non-defined residues or with non-specified number of repetitions for structural elements in our study. Nevertheless, our results are plausible and comparable with online based workflows using HMDB and MassTRIX. After entering workflow step C2.1/C2.2 with the resulting sum formulas from HMDB or MassTRIX, followed by pathway mapping, all pathways identified in our workflow were covered. Repeating the evaluation step C2.1/C2.2 was necessary because the web databases only use mass error for identification, while local processing in our workflow also considers the isotopic pattern, and thus reduces the number of false-positive annotations (see Table 2). A comparison of online and offline evaluations by Lokhov et al. (2020) also resulted in marked differences in number of annotated metabolites. Another aspect to be considered is the occasional lack of updated links to recent databases used in the web-based tools.

Another important quality parameter to reduce the number of false annotations, was pathway coverage, which was calculated based on sum formulas. Here we followed the conservative procedure according to Denihan et al. (2019) by counting isobaric sum formulas only once if there were multiple present in one pathway. This avoided overrepresentation of isobaric compounds and thus extreme effects on pathway coverage. In addition, we used two further quality parameters: 3 linked non-isobaric metabolites and unique sum formulas for the respective pathway. These quality parameters reduced the number of identified pathways from 83 to 71. DI-FT-ICR-MS measurements, paired with pathway enrichment, have been used by Witting et al. (2015); Shahbazy et al. (2020), and Denihan et al. (2019), but with web-based approaches and with neither used as a filtering element. According to current knowledge, filtering based on the specified quality parameters (see 2.2.5/2.2.7) is a new approach to eliminate false-positive annotations.

Further, evaluating the co-existence of isobaric compounds was improved for the first time using pathway enrichment analyses. This step allowed us to analyze the plausibility of metabolite annotations by integrating them into a pathway and directly linking metabolites to the biological context. Thus, in the pentose phosphate pathway, the co-existence of the two isobars fructose-6-phosphate and glucose-6-phosphate was verified. In addition, the occurrence of 3-ureidoisobutyrate could be excluded, whereas the occurrence of the isobaric compound glutamine was confirmed by alanine, aspartate, and glutamate metabolism. However, there are also limitations; terminal isobaric compounds of a pathway with the same precursor molecules cannot be further evaluated for their presence without additional analysis. The same applies to the occurrence of isobaric compounds with precursor molecules and degradation products that are also isobaric compounds.

### 4.2 Caco-2 metabolome

The Caco-2 cell metabolome described here shows the direct microbiome independent effect of the used non-digestible oligosaccharides. *In vivo*, intestinal epithelial cells are the interface between host and microbiome and are exposed to many microenvironmental metabolites (Zaurito and Tschurtschenthaler, 2022). Non-digested oligosaccharides and resistant starches, consumed with food, largely reach the colon and are metabolized by the associated microbiota. The main fermentation products are short-chain fatty acids such as butyrate, acetate and propionate, and the disaccharide lactate. Butyrate for example serves as the main energy source for colonocytes (Salvi and Cowles, 2021). Previous studies by Kuntz et al. (2008), Zenhom et al. (2011), and Akbari et al. (2017) showed that oligosaccharides can also have a microbiome-independent effect, but focused on growth, proliferation and inflammatory responses and not on metabolomic changes.

To our best knowledge, the present study is the first characterization of the Caco-2 metabolome in response to galactooligosaccharide and milk oligosaccharide exposures. There is a comparable approach to Caco-2 cells and inulin regarding metabolomic fingerprinting, by Lamers et al. (2003), but this only referred to individual metabolites, not pathways. Another study by Lafontaine et al. (2020) evaluated the response of CaCo-2 cells to GOS and FOS (fructo-oligosaccharides) on the pathway transcriptome level. Thus, there is a clear need for more metabolome research (Del Fabbro et al., 2020).

Based on the workflow, it was possible to identify 71 active pathways that characterize the treatment-specific Caco-2 metabolome. These pathways led to the identification of 19 hub metabolites that occurred in at least 6 of these metabolic pathways. Pairwise comparisons of the treatments resulted in identification of 275 significantly regulated metabolites and 25 significantly regulated pathways.

The pathways that describe the Caco-2 metabolome were divided into 12 main categories, and a number of minor categories with one pathway each. Carbohydrate metabolism and amino acid metabolism were the most important categories (Figure 2). This was expected, because carbohydrates are the most abundant class of biomolecules, with central importance for energy metabolism. Amino acids are similarly essential, as central building blocks of proteins. The pathway category “cancer” reflects the origin of the cell line used, cultured from a carcinoma. Caco-2 cells are human colorectal adenocarcinoma cells (Fogh et al., 1977), which morphologically and functionally correspond to epithelial cells of the small intestine in the differentiated state (Pinto et al., 1983). The main function of enterocytes is the enzymatic digestion of food compounds, which explains the significance of the “digestive system” and “lipid metabolism” categories. Growth is accompanied by cell division, which is particularly pronounced in cancer cells. Cell division is associated with DNA replication, and DNA of course consists of nucleotides, which provides the link to “nucleotide metabolism”. In addition to digestive enzymes, Caco-2 cells also possess cytochrome P450 enzymes, which explains the presence of “xenobiotics biodegradation and metabolism” category (Rosenberg and Leff, 1993; Meunier et al., 1995). The cultivation medium also contained vitamins, which epithelial cells and Caco-2 cells are able to take up *via* various transporters and passive diffusion (Dix et al., 1990; Albersen et al., 2013). In this context, the presence of degradation products attributable to the “metabolism of cofactors and vitamins” category is plausible.

#### 4.2.1 Pentose phosphate metabolism and fructose and mannose metabolism

We showed a significant regulation of pentose phosphate metabolism (C/LGM vs*.* HG) and fructose and mannose metabolism (C vs. HG). Caco-2 cells are able to transfer glucose from the surrounding medium into the cell *via* the GLUT1 or SGLT1 transporters (Mahraoui et al., 1992; Mahraoui et al., 1994; Bissonnette et al., 1996). The *p*-values of the hub metabolites indicate that the content of hexoses had the highest concentration in the controls and the LGM treatment (see Table 3). It is known that in the case of an excess of glucose, glucose enters into pentose phosphate metabolism *via* glucose-6-P, as a side arm of glycolysis. In addition to the pentoses or riboses for the synthesis of nucleotides, pentose phosphate metabolism is the main source of NADPH. NADPH is one of the most important coenzymes in many anabolic processes, but it is also central in the scavenging of reactive oxygen species (ROS) by contributing to the regeneration of oxidized glutathione (Patra and Hay, 2014). An increased cell division rate and the increased occurrence of ROS are characteristic of cancer cells compared to non-cancerous cells. In addition to pentose phosphate metabolism, excess glucose can also be used for polyol metabolism, which in turn yields fructose. In this context, glucose is converted to sorbitol by an aldose reductase and then to fructose by a sorbitol dehydrogenase. Pronounced polyol metabolism has previously been described for cancer cell lines, including Caco-2, and is related to the aggressiveness of tumors (Schwab et al., 2018). This could explain why fructose and mannose metabolism is significantly regulated in the control compared to the HG treatment, although no fructose or sucrose were present in the medium. Sorbitol was also detected in significantly higher amounts in the controls compared to the HG treatment, further supporting this theory.

#### 4.2.2 Degradation of trisaccharides

A significantly higher content of tri- and disaccharides was observed in the HG-treated cells (HG vs. C/GM/LGM). 6'-galactosyllactose is a neutral oligosaccharide, which can enter Caco-2 cells by transcellular transport *via* transcytosis (Gnoth et al., 2001, 2002). It is unclear why disaccharide accumulation occurred as they are usually cleaved into monosaccharides before uptake. It can be speculated that the terminal glucose of 6'-galactosyllactose was cleaved by disaccharidase, possessing the same glyosidic linkage as lactose, resulting in the accumulation of disaccharides.

#### 4.2.3 Amino acid metabolism and vitamin B6 uptake

Furthermore, a significantly higher content of various amino acids was observed in association with HG (HG vs. C/LGM/GM), including glutamate, glutamine, aspartate, and asparagine. An increase in glutamate in Caco-2 cells in the context of inulin treatment was also described by Lamers et al. (2003). Glutaminolysis is particularly pronounced in cancer cells as an alternative energy metabolism. Thereby glutamine is degraded to glutamate *via* glutaminase, which is in turn metabolized to alpha-ketoglutarate and supplied to the citrate cycle, resulting in malate. Malate was also detected in the HG-treated cells with significantly higher content compared to the control. Malate can cytosolically be degraded to pyruvate or, alternatively, *via* oxaloacetate, to aspartate and *via* this contribute to asparagine regeneration and nucleotide synthesis (Yang et al., 2017). The amino acids aspartate and asparagine, as well as the nucleoside cytidine, were detected in significantly higher amounts in the HG-treated cells. Furthermore, a significantly higher content of 5-oxo-proline and glutathione was detected. 5-oxo-proline is considered a storage form of glutamate and is involved in glutathione metabolism. This contrasts with the observations of Lafontaine et al. (2020), who described a downregulation of glutathione biosynthesis in the presence of GOS. In addition, a significantly higher metabolism of vitamin B6 was observed in the HG-treated cells. Phosphorylated vitamin B6 derivatives function as coenzymes in enzymatic reactions that are primarily related to the metabolism of amino acids (Parra et al., 2018). Since vitamin B6 was also part of the medium, it is possible that there is a correlation of increased amino acid uptake and vitamin B6.

#### 4.2.4 Amino acid transport and osmoregulation

The significantly higher amino acid content in the cells treated with HG may also be due to increased transport activity, as all amino acids were also part of the medium. Lafontaine et al. (2020) showed in transcriptome analyses after GOS treatment of Caco-2 cells, an increased expression of genes related to L-type amino acid transporters2. This could also explain the significant content of serine, threonine, and histidine, since threonine and histidine are essential amino acids. Interestingly, the accumulated amino acids are glucogenic or partially glucogenic, which is in line with the lower glucose content in the HG treatment. Furthermore, a significant upregulation of glycerophospholipid metabolism was observed in association with HG. These were primarily phosphocholines and phosphoethanolamines and their metabolites. Phospholipids are constituents of bio membranes and are crucial for cell structure, cell signaling, and energy metabolism (Dowhan, 2017). However, phosphoethanolamines and phosphocholines were explicitly degraded. This is possibly related to a release and therefore a significantly higher content of taurine in the HG-treated cells. Taurine is an osmoregulator influencing ionic fluxes within the cell (Schaffer et al., 2000). Many of the transport systems present in the cell function as Na^+^ symporters. It can be assumed that there is an excess of Na^+^ ions in the cell in connection with the increased uptake of amino acids. Taurine contributes to preventing this imbalance.

#### 4.2.5 Proton shuttle systems and scavenger molecules

Significantly higher levels of histidine and glycerol-3-phosphates were detected in all treatments containing GOS. This could be related to increased proton shuttle processes. The glycerol-3-phosphate shuttle system contributes to the transport of cytosolically produced NADH into the mitochondria, which is subsequently transferred to the respiratory chain for ATP production. The inner mitochondrial membrane is impermeable to NADH, so that a shuttle-dependent transport must take place (Purvis and Lowenstein, 1961). Histidine is also able to bind protons. The protonation takes place at the nitrogen of the imidazole side chain. The reaction partner becomes a nucleophile when originally uncharged or is neutralized when positively charged. Often other amino acids are part of this reaction (Ingle, 2011). We assume that the increased proton shuttle processes are related to the GOS-dependent increase in glutaminolysis and uptake of amino acids. In contrast, histidine may function as a scavenger molecule. Son et al. (2005) showed for Caco-2 cells a dose-dependent inhibition of hydrogen peroxide- and TNF-α-induced IL-8 secretion. Therefore, histidine could be considered a systemic cellular response to GOS treatment and thus be related to the anti-inflammatory potential of galactooligosaccharides.

#### 4.2.6 Cell signaling *via* second messenger

Significantly higher levels of the second messenger 1-myo-inositol-1,4,5-triphosphate (IP3) were observed in GM-treated cells (GM vs. C/HG/LGM). Thus, the present response must be based on cell surface sensing. The regulation could be related to the sialyllactose present in the bMOS fraction. Sialyllactose is an acidic oligosaccharide due to its side chain. Gnoth et al. (2001) showed that acidic oligosaccharides are transported only *via* paracellular transport in Caco-2 monolayers. As described by Triantis et al. (2018), galectins may be involved. Within the cell, IP3 contributes to the increased release of calcium ions from the endoplasmic reticulum and to numerous calcium-dependent responses, including cell proliferation and cell signaling (Berridge, 1995).

## 5 Conclusion

The established local workflow offers an alternative to web-based approaches. It considers isotope pattern as well as mass error, additional quality parameters that lead to higher precision in identification and annotation. By incorporating the biological context through the pathway enrichment analyses and the criteria established for the enrichment analyses, it is possible to evaluate the co-presence of isobars. A further advantage of the local workflow is its independence and fast adaptability. The entire workflow can be applied to other cell culture systems and organisms as well as to various treatments and measurement techniques, whereby isotope fine structure can only be used as a quality parameter for xHR instruments.

Applying the presented workflow allows a detailed characterization of the Caco-2 metabolome and forms the basis for further investigations of bioactive compounds in cell culture experiments.

Moreover, it was shown for the first time that GOS and MOS can directly, i.e., independently of the microbiome, affect the metabolome of Caco-2 cells, ranging from single metabolites to the regulation of entire pathways. Most importantly amino acid pathways (D-glutamine and D-glutamate metabolism and histidine metabolism) were upregulated by GOS vs. the other treatments.

## Data Availability

The original contributions presented in the study are included in the article/Supplementary Material, further inquiries can be directed to the corresponding author.
